# Mitochondrial complex 1 gene analysis in keratoconus

**Published:** 2011-06-08

**Authors:** Dhananjay Pathak, Bhagabat Nayak, Manvendra Singh, Namrata Sharma, Radhika Tandon, Rajesh Sinha, Jeewan S. Titiyal, Rima Dada

**Affiliations:** 1 Laboratory For Molecular Reproduction and Genetics, Department of Anatomy, All India Institute of Medical Sciences, Ansari Nagar, New Delhi, India; 2 Dr. R.P. Centre for Ophthalmic Sciences, All India Institute of Medical Sciences, Ansari Nagar, New Delhi, India; 3 Centre for Cellular and Molecular Biology, Uppal Road-500007, Hyderabad, Andhra Pradesh, India

## Abstract

**Purpose:**

Keratoconus is characterized by the thinning of corneal stroma, resulting in reduced vision. The exact etiology of keratoconus (KC) is still unknown. The involvement of oxidative stress (OS) in this disease has been reported. However, the exact mechanism of OS in keratoconus is still unknown. Thus we planned this study to screen mitochondrial complex I genes for sequence changes in keratoconus patients and controls, as mitochondrial complex I is the chief source of reactive oxygen species (ROS) production.

**Methods:**

A total of 20 keratoconus cases and 20 healthy controls without any ocular disorder were enrolled in this study. Mitochondrial complex I genes (ND1, 2, 3, 4, 4L, 5, and 6) were amplified in all patients and controls using 12 pairs of primers by PCR. After sequencing, DNA sequences were analyzed against the mitochondrial reference sequence NC_012920. Haplogroup frequency based Principle Component Analysis (PCA) was constructed to determine whether the gene pool of keratoconus patients is closer to major populations in India.

**Results:**

DNA sequencing revealed a total 84 nucleotide variations in patients and 29 in controls. Of 84 nucleotide changes, 18 variations were non-synonymous and two novel frame-shift mutations were detected in cases. Non-synonymous mtDNA sequence variations may account for increased ROS and decreased ATP production. This ultimately leads to OS; which is a known cause for variety of corneal abnormalities. Haplotype analysis showed that most of the patients were clustered under the haplogroups: T, C4a2a, R2’TJ, M21’Q1a, M12’G2a2a, M8’CZ and M7a2a, which are present as negligible frequency in normal Indian population, whereas only few patients were found to be a part of the other haplogroups like U7 (Indo-European), R2 and R31, whose origin is contentious.

**Conclusions:**

Mt complex I sequence variations are the main cause of elevated ROS production which leads oxidative stress. This oxidative stress then starts a cascade of events which ultimately can lead to keratoconus. Prompt antioxidant therapy should be initiated in keratoconus patients to minimize ROS related damage.

## Introduction

Keratoconus (KC; Mendelian Inheritance in Man [MIM] 14830) is a rare disease characterized by thinning of the corneal stroma, resulting in reduced vision, irregular astigmatism and corneal scarring. The age of onset is mostly at puberty with the estimated prevalence of KC is 1–4 in 2000 in general population. Exact cause of KC is still unclear, although pathogenesis may involve genetic [1,2], environmental and behavioral factors [3,4]. KC is one of the most common cause for corneal transplantation. Inheritance of KC does not usually follow a simple Mendelian inheritance in majority of cases and appears to be sporadic [5], but positive family history is reported in 6% to 10% of patients [6]. Both recessive and dominant pattern of inheritance have been reported with predominance of autosomal dominant inheritance and disease shows incomplete penetrance and variable expressivity [7,8]. Since, cornea is an avascular structure and first target of ultraviolet radiation entering the eyes, it is potentially vulnerable to oxidative stress [9]. Studies report the role of oxidative damage in KC and other corneal diseases [10,11].

In a previous study from our laboratory we have reported association of mtDNA variations with congenital glaucoma [12]. We have shown the presence of mtDNA complex I sequence variations which were associated with elevated ROS production in congenital glaucoma. In this pilot study we reported the results of mitochondrial complex I gene analysis in 20 keratoconus patients negative for Visual System Homeobox 1 (*VSX1*) mutations [13].

## Methods

### Ethics statement

All patients provided informed consent before participation in this study. The study was approved by Institutional Review Board (IRB00006862; All India Institute of Medical Sciences, New Delhi, India).

### Clinical examination and selection of cases

A total of twenty keratoconus patients (Table 1) presented (during April 2009 to April 2010) at the Dr. R. P. Centre for Ophthalmic Sciences (AIIMS, New Delhi, India) were enrolled in this study. Clinical evaluation involved Ultrasonic Pachymetry, videokeratography (VKG), Orbscan, visual testing, fundoscopy, slitlamp-biomicroscopy, and retinoscopy. Of these patients, 14 were males and 6 were females. The mean age of presentation was 17.2 years. Diagnosis of keratoconus involved the presence of characteristic topographic features, such as inferior or central corneal steepening, or an asymmetric bowtie pattern with skewing of the radial axes, and the presence of one or more of the following characteristic, clinical features in one or both eyes: conical corneal deformation, munsen sign, corneal stromal thinning, a Fleischer ring or Vogt striae. Family history up to three generations was collected and pedigrees were drawn. All 20 cases were sporadic without any family history. All keratoconus cases secondary to causes like trauma, surgery, Ehlers Danlos syndrome, osteogenesis imperfecta, and pellucid marginal degeneration were excluded from the study. Twenty ethnically matched normal individuals without any ocular disorder were enrolled as controls. Health information was obtained from controls through the questionnaire; all underwent ophthalmological examination. Five milliliters of blood was collected by venipuncture in EDTA (EDTA) vaccutainers (Greiner Bio-One GmbH, Frickenhausen, Germany) from both patients and controls. DNA was extracted from whole blood samples by the inorganic method. For the population study, controls were taken from published data defining the lineage of the Indian population [14,15].

**Table 1:** Clinical phenotype of keratoconus patients.

**Patient ID**	**Age in years**	**Sex**	**Visual acuity in Snellen’s chart**		**Munsen sign**		**Vogt's striae**		**Hydrops**		**Scarring**		**Keratometry in VKG (in diopters)**		**Ultrasonic pachymetry (in micrometers)**	
			OD	OS	OD	OS	OD	OS	OD	OS	OD	OS	OD	OS	OD	OS
KC1	20	F	6/12	6/12	+	+	-	-	-	-	-	-	45.62	46.37	490	414
KC2	12	M	6/60	6/60	+	+	+	+	+	+	+	+	56	52	344	347
KC3	22	F	6/12	6/6	+	+	+	+	+	-	+	-	56.6	49.5	396	412
KC4	20	M	6/12	6/9	+	+	+	+	+	-	+	-	48.5	45	406	498
KC5	20	M	6/24	6/6	+	-	+	-	+	-	+	-	54	46.5	410	520
KC6	19	M	6/12	6/12	+	+	+	+	-	-	-	-	46.5	46.5	501	488
KC7	18	M	618	6/60	+	+	+	+	-	+	-	+	52.12	Distorted	480	344
KC8	14	F	6/36	6/24	+	+	+	+	+	+	+	+	>52	52	336	346
KC9	20	M	6/9	6/9	+	+	+	+	-	-	-	-	50.5	49.1	460	436
KC10	22	M	6/24	6/9	+	-	+	-	-	-	-	-	52	47.1	344	420
KC11	19	M	6/18	6/18	+	+	+	+	+	+	+	-	54.75	56	410	400
KC12	17	M	6/12	6/18	+	+	+	+	-	-	-	-	51.12	49.87	419	414
KC13	20	F	6/9	6/12	+	+	+	+	-	-	-	-	48.5	51.25	440	456
KC14	10	M	6/12	6/9	+	+	+	-	+	-	+	-	61.12	48.75	402	512
KC15	22	F	6/12	6/12	+	+	-	+	-	-	+	+	48.5	48	502	486
KC16	18	F	6/6	6/60	-	-	-	+	-	+	-	+	Distorted	49.5	510	265
KC17	22	M	6/6	6/9	+	+	+	+	-	-	-	-	49.5	51.75	399	353
KC18	15	M	6/9	6/18	+	+	+	+	+	-	-	-	48.25	48.75	485	493
KC19	20	F	6/60	6/60	+	+	+	+	+	+	+	+	Distorted	Distorted	230	330
KC20	16	M	6/12	6/9	+	+	+	+	-	-	-	-	48	48.25	484	496

Key: M=male; F=female; OD=right eye; OS=left eye; +=positive; -=negative; VKG=videokeratography.

### Polymerase chain reaction (PCR) amplification and sequence analysis of the mitochondrial DNA coding region

The mitochondrial complex 1 (*ND1*, *ND2*, *ND3*, *ND4*, *ND4L*, *ND5*, and *ND6* [ND stands for NADH dehydrogenase]) was amplified in all patients and controls using 12 pairs of primers using cycling conditions as described by Kumar and associates [16] and presented in Table 2. Briefly, PCR amplifications for all primer sets were performed in a 40-μl volume containing 1.0 μl of 20 μM stock solution for each primer (Eurofins Genomics India pvt Ltd, Bangalore, India), 100 ng of genomic DNA, 1 unit of Taq polymerase (Banglore Genei, Bengaluru, Karnataka, India), 0.1 mM of each deoxynucleotide triphosphate (dNTP), and 4 μl of 10× PCR buffer (with 15 mM MgCl_2_) by means of 30 cycles of amplification, each consisting of 30 s denaturation at 94 °C, 30 s annealing at 55 °C, and 1 min extension at 72 °C. Finally, an extension for 5 min at 72 °C was performed. Amplified PCR products were purified using a gel/PCR DNA fragments extraction kit (catalog number DF100; Geneaid Biotech Ltd., Sijhih City, Taiwan). Purified PCR products of both primers (forward and reverse) were sent for sequencing to MCLAB (Molecular Cloning Laboratories, South San Francisco, CA). All sequence variants from both KC patients and controls were compared to the Human Mitochondrial reference sequence NC_012920 provided by the National Center for Biotechnology Information (NCBI) using ClustalW2 (multiple sequence alignment program for DNA; European Molecular Biology Laboratory (EMBL)-European Bioinformatics Institute (EBI).

**Table 2:** Primer used for amplification of complex-I gene of mitochondria.

**Primer name**	**Primer sequence**	**Product size**	**Melting temperature (°C)**
1	F-3’GGACTAACCCCTATACCTTCTGC5’		
	R-3’GGCAGGTCAATTTCACTGGT5’	859	55
2	F-3’AAATCTTACCCCGCCTGTTT5’		
	R-3’AGGAATGCCATTGCGATTAG5’	885	55
3	F-3’TACTTCACAAAGCGCCTTCC5’		
	R-3’ATGAAGAATAGGGCGAAGGG5’	831	55
4	F-3’TGGCTCCTTTAACCTCTCCA5’		
	R-3’AAGGATTATGGATGCGGTTG5’	903	55
5	F-3’ACTAATTAATCCCCTGGCCC5’		
	R-3’CCTGGGGTGGGTTTTGTATG5’	978	55
6	F-3’TCTCCATCTATTGATGAGGGTCT5’		
	R-3’AATTAGGCTGTGGGTGGTTG5’	892	55
7	F-3’GCCATACTAGTCTTTGCCGC5’		
	R-3’TTGAGAATGAGTGTGAGGCG5’	859	55
8	F-3’TCACTCTCACTGCCCAAGAA5’		
	R-3’GGAGAATGGGGGATAGGTGT5’	801	55
9	F-3’TATCACTCTCCTACTTACAG5’		
	R-3’AGAAGGATATAATTCCTACG5’	865	55
10	F-3’AAACAACCCAGCTCTCCCTAA5’		
	R-3’TCGATGATGTGGTCTTTGGA5’	976	55
11	F-3’ACATCTGTACCCACGCCTTC5’		
	R-3’AGAGGGGTCAGGGTTGATTC5’	969	55
12	F-3’GCATAATTAAACTTTACTTC5’		
	R-3’AGAATATTGAGGCGCCATTG5’	937	55

### Computational assessment of missense mutations

For prediction of pathogenic characteristics of all non-synonymous mtDNA changes two homology based programs PolyPhen-2 (Polymorphism Phenotyping) and SIFT (Sorting Intolerant From Tolerant) were used.

PolyPhen structurally analyzes an amino acid polymorphism and predicts whether that amino acid change is likely to be deleterious to protein function [17-19]. Polyphen-2 is more advanced version of the earlier version PolyPhen [20]. The prediction is based on the position-specific independent counts (PSIC) score derived from multiple sequence alignments of observations in case of functional domain of protein and predicted hydrophobic and transmembrane (PHAT) matrix element difference in case of transmembrane region of protein. PolyPhen scores of above 0.85 indicate the polymorphism is probably damaging to protein function. Scores of above 0.15 are possibly damaging, and scores of less than 0.15 are classified as benign.

SIFT is a sequence homology-based tool that sorts intolerant from tolerant amino acid substitutions and predicts whether an amino acid substitution in a protein will have a phenotypic effect [21-23]. SIFT is based on the premise that protein evolution is correlated with protein function. Positions important for function should be conserved in an alignment of the protein family, whereas unimportant positions should appear diverse in an alignment. Positions with normalized probabilities less than 0.05 are predicted to be deleterious and, those greater than or equal to 0.05 are predicted to be tolerated.

### Haplogroup and phylogenetic analysis

To check the fidelity of our conclusion, the evolutionary information and the significance of mutations should be known. For haplogroups (Hg) analysis we have carefully chosen two hundred healthy individual samples from same area for comparison analysis and these were also treated as controls. For all control samples, sequences of the control region were determined from position 16024 to 00300, using the ABI Prism Dye Terminator cycle-sequencing protocols developed by Applied Biosystems (Perkin-Elmer, Foster City, CA), to provide an initial presumed Hg assignment and cases were haplogrouped by complete coding region sequences. The C-track length variation at positions 16182 and 16183 in HVS-I and the indels at positions 00309 and 00315 in HVS-II were excluded from further analyses. Hg assignment was then confirmed, based on control and coding region Hg defining polymorphisms determined by means of direct sequencing.

The NETWORK 4.5.1.6 program was run for placing all the mutations of control samples in their respective phylogenetic tree using the protocol as described at the Fluxus Engineering Website. The matrilineal lineages of the case were drawn manually in the reduced median network of control samples, to create the topology map we have applied the reduced median algorithm (r=1), followed by the median-joining algorithm (epsilon=2).

### Principal component analysis

To minimize errors both strands were double-sequenced. Principal component analysis (PCA) of mtDNA was performed. For this experiment we compiled our data of mitochondrial haplogroups. Controls samples of five different populations were taken from published data defining Indian lineage for comparative analysis representing each sector of India i.e., Northern India, North-Western India, Western India, Eastern India, and Southern India.

The MVSP software package (Kovach WL, Services KC. MVSP - A multi-variate statistical package for Windows ver 3.13m. 2004) was used to identify the principal components (PCs) of mitochondrial variations that lead to form a haplogroup for every individual. To express the relative importance of top two eigenvectors in the resulting PCA plot, two axes were scaled by a factor equal to the square root of the corresponding eigen value. This experiment was repeated to confirm the outcomes.

## Results

### Sequence variation in Complex I genes

DNA sequencing of Complex I genes revealed a total 84 nucleotide variation in patients (Table 3) and 29 variations in controls (Table 4). Of the 84 nucleotide variations in patients, 18 (21.42%) were non synonymous (Table 5), 52 (61.90%) were synonymous, 9 (10.71%) variations were in RNA genes and 3 (3.57%) were in non-coding region. Of 84 nucleotide variations in KC patients; five variations (3918G>A, 5348C>T, 12007G>A, 12372G>A and 12561G>A) were also present in controls. Out of 29 nucleotide variations found in controls 5 were non-synonymous. Maximum nucleotide variations were in *ND5* (n=28) followed by *ND4* (n=15); *ND2* (n=13); *ND3* (n=7); *ND1* (n=3); *ND4L* (n=3), tRNAs (n=9), rRNAs (n=2) and 3 in non-coding regions.

**Table 3:** Complex 1variations observed in KC patients.

**Sample number**	**Genomic position**	**Base change**	**Gene/ location**	**Amino acid position**	**Codon change**	**Amino acid change**	**Change in protein**	**Total no. of patients with nt changes**	**GeneBank accession number if novel**
1	2706	G>A	16rRNA					1	
2	2887	T>C	16rRNA					1	
3	3918	G>A	ND1	204	GAG>GAA	Glu>Glu	p.E204E	1	
4	3921	T>C	ND1	205	TCT>TCT	Ser>Ser	p.S205S	1	
5	4216	T>C	ND1	304	TAT>CAT	Tyr>His	p.Y304H	3	
6	4454	T>C	tRNA met					1	
7	4682	C>A	ND2	71	CTA>ATA	Leu>Ile	p.L71I	1	Yes
8	4688	T>C	ND2	73	GCT>GCC	Ala>Ala	p.A73A	1	
9	4696	T>C	ND2	76	TTC>TCC	Phe>Ser	p.F76S	1	Yes
10	4715	A>G	ND2	100	ATG>ATA	Met>Met	p.M100M	1	
11	4833	A>G	ND2	122	ACC>GCC	Thr>Ala	p.T122A	1	
12	4917	A>G	ND2	150	AAC>GAC	Asn>Asp	p.N150D	1	
13	5046	G>A	ND2	193	GTT>ATT	Val>Ile	p.V193I	1	
14	5108	T>C	ND2	213	ACT>ACC	Thr>Thr	p.T213T	1	
15	5300	T>T, Del CA	ND2	277	Frame shift	p.Ile277His fs X11	p.I287X	1	Yes
16	5348	C>T	ND2	293	TAT>TAC	Tyr>Tyr	p.Y293Y	1	
17	5351	A>G	ND2	294	TTA>TTG	Leu>Leu	p.L294L	1	
18	5360	C>T	ND2	297	ATC>ATT	Ile>Ile	p.I297I	1	
19	5460	G>A	ND2	331	GCC>ACC	Ala>Thr	p.A331T	1	
20	5580	C>T	NC					20	
21	5585	G>A	NC					20	
22	5601	C>T	tRNA Ala					2	
23	5790	C>A	OL					1	
24	5899	InsC	NC					1	Yes
25	5900	InsC	NC						Yes
26	10084	T>C	ND3	9	ATC>ACC	Ile>Thr	p.I9T	1	
27	10142	C>T	ND3	18	AAC>AAT	Asn>Asn	p.N18N	1	
28	10253	T>C	ND3	65	TTT>TTC	Phe>Phe	p.F65F	1	
29	10304	T>C	ND3	82	ACT>ACC	Thr>Thr	p.T82T	1	Yes
30	10373	G>A	ND3	105	GAG>GAA	Glu>Glu	p.E105E	1	
31	10398	G-A	ND3	114	GCC-ACC	Ala>Thr	p. A 114 T	8	
32	10400	C-T	ND3	114	GCC-GCT	Ala-Ala	p. A114 A	9	
33	10411	A>G	tRNA Arg						
34	10463	T>C	tRNA Arg					1	
35	10685	G>A	ND4L	72		Ala>Ala	p.A72A	1	
36	10631	C>T	ND4L	54		Leu>Leu	p.L54L	1	
38	10819	A>G	ND4	20	CTG>CTA	Lys-Lys	p.K20K	20	
39	10873	C-T	ND 4	38	CCC-CCT	Pro-Pro	P38P	20	
40	10951	C>T	ND4	64	CCC>CCT	Pro>Pro	p.P64P	1	Yes
41	11017	C-T	ND 4	86	AGC-AGT	Ser-Ser	S86S	20	
42	11251	A>G	ND4	164	CTA>CTG	Leu>Leu	p.L164L	1	
43	11273	G>C, Del G	ND4	172	Frame shift	p.gly172AlafsX2	p.L174X	1	Yes
44	11437	T>C	ND4	226	GCT>GCC	Ala>Ala	p.A226A	1	
45	11467	A>G	ND4	236	TTA>TTG	Leu>Leu	p.L236L	3	
46	11673	C>T	ND4	305	ACC>ACT	Thr>Thr	p.T305T	1	
47	11722	C>T	ND4	321	CTC>CTT	Leu>leu	p.L305L	20	
48	11902	G>C	ND4	381	GTG>GTC	Val>Val	p.V381V	1	
49	11914	G>A	ND4	385	ACG>ACA	Thr>Thr	p.T385T	1	
50	11947	A>G	ND4	396	ACA>ACG	Thr>Thr	p.T396T	1	
51	11969	G>A	ND4	404	GCC>ACC	Ala>thr	p.A404T	3	
52	12007	G>A	ND 4	416	TGG-TGA	Tryp-Tryp	p. W416 W	9	
53	12234	G>A	tRNA Ser					1	
54	12236	G>A	tRNA Ser					1	
55	12330	A>G	tRNA Ser					3	Yes
56	12308	A>G	tRNA Leu					5	
37	12330	A>G	tRNA Leu						
57	12361	A>G	ND5	9	ACC>AAA	Thr>Ala	p.T9A	1	
58	12372	G>A	ND5	12	CTG>CTA	Leu>Leu	p.L12L	5	
59	12414	T>C	ND5	26	CCT>CCC	Pro>Pro	p.P26P	1	
60	12426	C>A	ND5	30	AAC>AAA	Asp>Lys	p.D30L	1	
61	12561	G>A	ND5	75	CAG>CAA	Gln>Gln	p.Q75Q	1	
62	12633	C>A	ND5	99	TCC>TCA	Ser>Ser	p.S99S	2	
63	12624	T>C	ND5	96	GTT>GTC	Val>Val	p.V96V	1	
64	12672	A>G	ND5	112	CCA>CCG	Pro>Pro	p.P112P	2	
65	12654	A>G	ND5	106	TGA>TGG	Trp>Trp	p.W106W	1	Yes
66	12705	T-C	ND5	123	ATT-ATC	Ile-Ile	p. I 123 I	11	
67	12850	G-A	ND 5	172	GTC-ATC	Val –Ile	p. I 172 V	20	
68	12879	G>C	ND5	181	GGT>GGC	Gly>Gly	P.G181G	1	
69	13065	C>T	ND5	243	GTC>CTT	Val>Val	p.V243V	1	
70	13104	A>G	ND5	256	GGA>GGG	Gly>Gly	p.G256G	1	
71	13174	T>C	ND5	280	TTA>CTA	Leu>Leu	p.L280L	1	
72	13263	A>G	ND5	309	CAA>CAG	Gln>Gln	p.Q309Q	1	
73	13368	G>A	ND5	344	GGG>GGA	Gly>Gly	p.G344G	2	Yes
74	13434	A>G	ND5	367	ATA>ATG	Met>Met	p.M366M	2	
75	13488	T>C	ND5	384	GGT>GGC	Gly>Gly	p.G384G	1	
76	13500	T>C	ND5	388	GGT>GGC	Pro>Pro	p.P388P	3	
77	13563	A>G	ND5	409	CTA>CTG	Leu>Leu	P.L409L	4	
78	13557	A>G	ND5	407	TGA>TGG	Trp>Trp	p.W407W	1	Yes
79	13617	T>G	ND5	427	ATT>ATC	Ile>Ile	p.I427I	2	
80	13637	A>G	ND5	434	CAA>CAG	Glu>Arg	p.Q434R	2	
81	13768	T>C	ND5	478	TTC>ATC	Phe>Ile	p.F479I	3	
82	13914	C>A	ND5	526	CTC>CTA	Leu>Leu	p.L526L	3	
83	14058	C>T	ND5	574	TCC>TCT	Ser>Ser	p.S574S	1	
84	14070	A>G	ND5	578	TCA>TCG	Ser>Ser	p.S578S	1	

Abbreviations: KC represents Keratoconus; nt- Nucleotides, A – Adenine; T – Thymine; G- Guanine; C- Cytosine; ND1 represents NADH dehydrogenase subunit 1, ND2 represents NADH dehydrogenase subunit 2; ND3- NADH dehydrogenase subunit 3; ND4- NADH dehydrogenase subunit 4; ND4L- NADH dehydrogenase subunit 4L; ND5- NADH dehydrogenase subunit 5.

**Table 4:** Complex 1 variations observed in controls.

**Sample number**	**Genomic position**	**Base change**	**Locus**	**Amino acid position**	**Codon change**	**Amino acid change**	**Change in protein**
1	3591	G>A	ND1	95	CTG>CTA	Thr>Thr	p.T95T
2	3915	G>A	ND1	203	GGG>GGA	Gly>Gly	p.G203G
3	3918	G>A	ND1	204	GAG>GAA	Glu>Glu	p.E204E
4	3933	A>G	ND1	209	TCA>TCG	Ser>Ser	p.S209S
5	3970	C>T	ND1	222	CTA>TTA	Leu>Leu	p.L222L
6	3996	C>T	ND1	230	AAC>AAT	Asn>Asn	p.N230N
7	4093	A>G	ND1	263	ACC>GCC	Thr>Ala	p.T263A
8	4029	C>A	ND1	241	ATC>ATA	Ile>Ile	p.I241I
9	4793	A>G	ND2	108	ATA>ATG	Met>Met	p.M108M
10	4852	T>A	ND2	128	CTG>CAG	Leu>Gln	p.L128Q
11	5186	A>T	ND2	239	TGA>TGT	Trp>Cys	p.W239C
12	5348	C>T	ND2	293	TAC>TAT	Tyr>Tyr	p.Y293Y
13	10310	G>A	ND3	84	CTG>CTA	Thr>Thr	p.T84T
14	11467	A>G	ND4	236	TTA>TTG	Leu>Leu	p.L236L
15	11914	G>A	ND4	385	ACG>ACA	Thr>Thr	p.T385T
16	12007	G>A	ND4	416	TGG>TGA	Trp>Trp	p.W416W
17	12073	C>T	ND4	438	TTC>TTT	Phe>Phe	p.F438F
18	12107	C>T	ND4	449	CTC>CTT	Thr>Thr	p.T449T
19	12133	C>T	ND4	458	TCC>TCT	Ser>Ser	p.S458S
20	13299	A>G	ND5	321	CAA>CAG	Gln>Gln	p.Q321Q
21	12372	G>A	ND5	12	CTG>CTA	Tyr>Tyr	p.T12T
22	12373	A>G	ND5	13	ACT>GCT	Thr>Ala	p.T13A
23	12406	G>A	ND5	24	GTT>ATT	Val>Ile	p.V24I
24	12486	C>T	ND5	50	CCC>CCT	Pro>Pro	p.P50P
25	12498	C>T	ND5	54	TTC>TTT	Phe>Phe	p.F54F
26	12561	G>A	ND5	75	CAG>CAA	Gln>Gln	p.Q75Q
27	13731	A>G	ND5	465	GGA>GGG	Gly>Gly	p.G465G
28	13806	C>T	ND5	490	GCC>GCT	Ala>Ala	p.A490A
29	14058	C>T	ND5	574	TCC>TCT	Ser>Ser	p.S574S

Abbreviations: A – Adenine; T – Thymine; G- Guanine; C- Cytosine; ND1 represents NADH dehydrogenase subunit 1, ND2 represents NADH dehydrogenase subunit 2;ND3- NADH dehydrogenase subunit 3; ND4-NADH dehydrogenase subunit 4; ND5- NADH dehydrogenase subunit 5.

**Table 5:** Mitochondrial DNA sequence variations (non-synonymous) detected in KC patients.

**Sample number**	**Nucleotide substitution**	**Amino acid change**	**Change in protein**	**SIFT score**	**SIFT prediction**	**Polyphen score**
2	4216T>C	Tyr>His	p.Y304H	0.82	Tolerant	0.008
3	4917 A>G	Asn>Asp	p.N150D	0.14	Tolerant	0.006
4	5046 G>A	Val>Ile	p.V193I	0.61	Tolerant	0.000
5	4833 A>G	Thr>Ala	p.T122A	0.10	Tolerant	0.135
6	4682 C>A	Leu>Ile	p.L71I	0.01	Pathogenic	0.001
7	4696 T>C	Phe>Ser	p.F76S	0.14	Tolerant	0.000
8	5300T>T	p.Ile277His fs X11	p.I287X			
9	5460 G>A	Ala>Thr	p.A331T	0.37	Tolerant	0.000
10	10084T>C	Ile>Thr	p.I9T	0.49	Tolerant	0.000
11	10398 G-A	Ala>Thr	p. A 114 T	1.00	Tolerant	0.000
12	11273G>C	p.gly172AlafsX2	p.L174X			
13	11969G>A	Ala>Thr	p.A404T	0.13	Tolerant	0.000
14	12361A>G	Thr>Ala	p.T9A	0.00	Pathogenic	Not available
15	12426C>A	Asn>Lys	p.N30L	0.21	Tolerant	0.899
16	12850G>A	Val >Ile	p. I172V	1.00	Tolerant	0.422
17	13637A>G	Glu>Arg	p.Q434R	0.41	Tolerant	0.008
18	13768T>C	Phe>Ile	p.F478I	0.17	Tolerant	0.005

**Figure 1:**
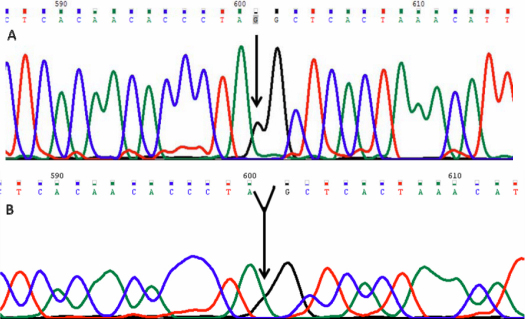
Mt DNA sequence chromatogram of *ND4* gene. **A**: The reference sequence derived from control is shown. **B**: Sequence derived from keratoconus patient K16 shows a deletion of G at11273, which cause a codon change GGC>GCT and p.gly172AlafsX2 (p.L174X) mutation.

Of 84 variations, 2 variations were frame-shift (11273G>A, 5300T>T). In one patient (KC 16) a single base deletion of guanine was observed at mtDNA position 11273. This caused a frame shift mutation after codon 172 (Gly>Ala) and introduced a stop codon at position 174 which resulted in a 173 amino acids truncated protein.This variation was homoplasmic (Figure 1).

**Figure 2:**
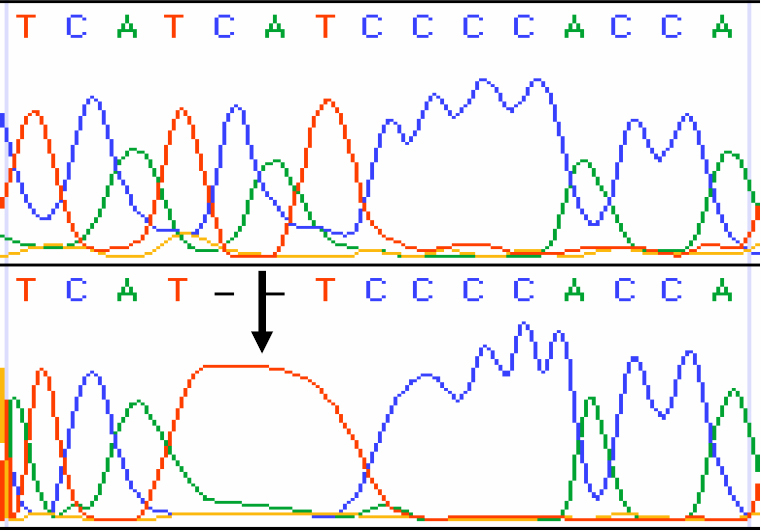
Mt DNA sequence chromatogram of *ND2* CA Deletion. **A**: The reference sequence derived from control is shown. **B**: Sequence derived from keratoconus patient K2 shows two base deletion CA at 5300, which causes a codon change ATC>ATT and p.Ile277His fsX11(p.I287X) mutation.

In patient KC 2, we found a 2 base pair (CA) deletion at genomic position 5300 and 5301. This frame shift mutation altered the amino acid reading frame in ND2 protein at position 277. This CA deletion produced a truncated protein of 287 amino acids (Figure 2).

### In silico analysis

SIFT analysis revealed two pathogenic changes (p.L71I and p.T9A) and PolyPhen revealed two pathogenic changes (p.N30L and p.I172V). The polyphen score of p.T9A was not available (no result for this mutation was available through PolyPhen; Table 5).

### Principle component analysis

**Figure 3:**
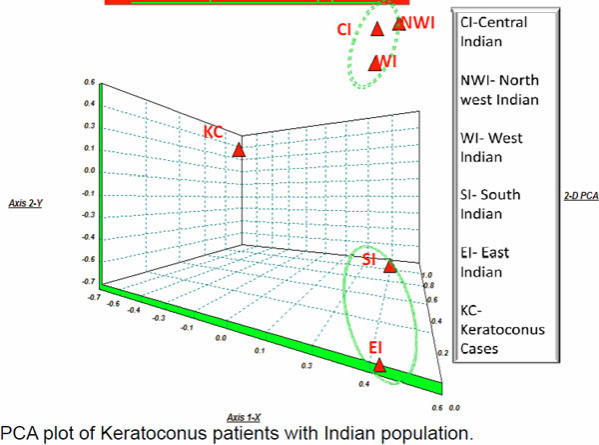
PCA plot of keratoconus patients with Indian population.

The tight cluster in Principal component Analysis (PCA) plot comprises the north-western, western, and north Indian population whereas the southern Indian and eastern Indian population is caught in a loose cluster (Figure 3). The controls were taken from published data defining Indian lineage for PCA and Haplogroup Network. We have treated the patients as a sub group of individuals having genetic structure different from normal Indians e.g., population. The inferences from PCA plot strongly supports our motive behind the planning of experiment, interestingly the patient population has not shown any relevant genetic affinity with other macropopulations of India.

## Discussion

In this study we analyzed mitochondrial complex 1 gene in 20 keratoconus patients (negative for VSX1 mutations [13]) and 20 unrelated healthy controls. The cornea, being an avascular structure and the first in line of ultraviolet (UV) radiation, is very susceptible to UV induced oxidative damage. Previous studies [10,11] suggested the role of oxidative stress in corneal disorders and congenital glaucoma [12]. Since the complex 1 NADH group of genes are most frequently associated with increased ROS production and oxidative stress [12,24], in this pilot study we analyzed mitochondrial complex I gene for sequence variations. Most of the mutations were found in *ND5* (n=28) followed by *ND4* (15) and then *ND2* (13). The frequent variations in *ND5* are in accordance with previous reports that mutations in *ND5* gene of complex 1 play an important role in mitochondrial diseases [25].

In this study we report two novel frame shift mutations. Patient (KC 2) harbored a two base deletion (CA) which caused a frameshift and introduced a stop codon at position 287 in protein (normal ND2 protein is 347 amino acids long). The truncated protein cannot substitute the wild type ND2 protein as frameshift altered the reading frame of *ND2*. Sequence variations in this gene are associated with several diseases e.g., Leigh syndrome, breast cancer, myocardial infarction, Parkinson disease, and primary congenital glaucoma (PCG) [12,26-29].

Patient (KC 16) harbored a single base deletion which resulted in a frame shift mutation after codon 172 (Gly>Ala) and introduced a stop codon at position 174 in protein and produced a truncated protein of 173 amino acids (wild type ND4 protein is 459 amino acids long).

Studies have documented that G10398A is associated with elevated ROS production due to altered complex 1 function [29-32]. Role of this allele G10398A has been implicated in diseases like congenital glaucoma, Parkinson, Type-2 diabetes, and in pre-term births [12,29-33]. The G10398A variation though associated with high ROS levels was present significantly higher in cases as compared to controls however this is present in 43% Indian population. The 4216T>C variation considered as secondary or intermediate LHON-Leber’s Hereditary optic neuropathy mutation was also present in 3 patients. However these patients had no features of LHON.

### Evolutionary insight of Mt complex I sequence variations

**Figure 4:**
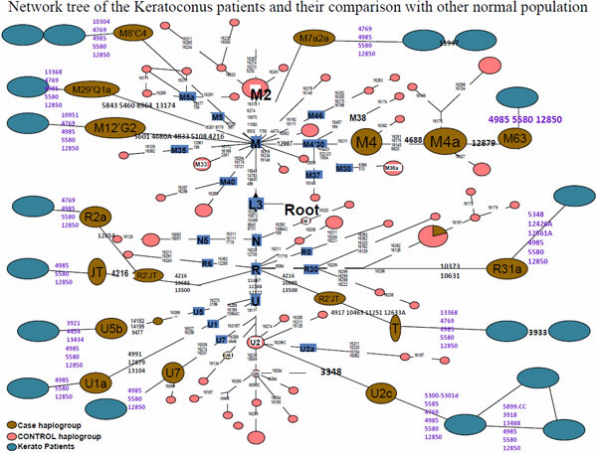
Network tree of the keratoconus patients and their comparison with other normal population.

The genetic diversity in India is very complex. Several mutations from even control regions have been classified into the associative agent for various diseases [34]. The degree of haplotype sharing between populations is to investigate the combined frequency of the shared haplotypes in two population groups. Thus, among the northern and the southern population groups the combined frequency of the haplotypes present also in the other group is significantly lower than that which we observed in the case of random groups. This is not surprising because West Eurasian-specific mtDNA haplogroups are rather frequent in northwest India [35]. Because the Indo-European and the Dravidic speakers of India are largely concentrated to the northern and southern parts of the subcontinent, respectively, the differences arising from geographic division of the Indian populations also correspond to different linguistic groupings [36]. In this study, we found that all the mutations were apparently North-Indian specific with some novel mutations. The sequencing of Complex 1 revealed 84 mutations, of which 14, including 2 frame shift mutations and 4 non-synonymous mutations, were novel and exclusively observed in KC patients. Interestingly, most of the patients and their maternal relatives were clustered under the haplogroups (T, C4a2a, R2’TJ, M21’Q1a, M12’G2a2a, M8’CZ, M7a2a, U5b1, U1a3) which are present as negligible frequency in normal Indian population, whereas only few patients were found to be a part of the haplogroups whose origin is contentious i.e., U7 (Indo-European), R2 and R31. We have found three patients who fall under Indian haplogroups (M4, M4’63, R31a1) but they also carry the same sets of novel synonymous and non-synonymous mutations i.e., 4769, 4985, 5580, and 12850 (Figure 4). We have found some novel mutations in addition to each individual’s lineages and they are different from each other. This finding suggests the positive/causative role of different combinations of the mitochondrial coding mutations in this disease, as the normal population, completely lack these mutations.

The patients harbored some novel mutations at the different sites in mitochondria i.e., 4769, 4985, 5580, and 12850. These variants have never been reported in any of the population studies whereas they were present in every patient. Nevertheless, it is impossible from the evolutionary point of view that these sets of mutations in the individuals from different haplogroup. By keeping in mind about mutation rates in the coding region and its natural selection [37], we propose that these variants could theoretically influence the patient’s phenotype. However, the variants present in coding regions of mitochondrial gene are not conserved in course of evolution. The patients were apparently homoplasmic (only one type of mtDNA was present). To determine whether the maternal inherited gene pool of keratoconus patients is truly closer to any major populations in India, we have constructed the haplogroup frequency based PCA plot for mtDNA (Figure 3). Indeed, this analysis shows ambiguously that the three Indian populations clusters tightly among themselves viz. North, North West and West populations and two populations are to be found in a loose cluster viz South Indian and East Indian, whereas the keratoconus population matches with none of them in the mtDNA PCA plots. However the genetic data indicates that the keratoconus patients comprise several different haplotypes, if they are compared to normal populations around them. Most of the patients are in the clades which are nonspecific to Indian lineages. This information suggests that keratoconus patients are among those who are recent migrants into India and some genes in mitochondria have acquired mutations which are not filtered by purifying selection. Our results explain that the patients are genetically unrelated to each other due to the present maternal lineages which were diversified in the history of evolution. This fact suggests that the polymorphisms which are playing pivotal roles in causing the disease are recently accumulated in the mitochondrial coding regions of an individual patient. We have found that mutations specifically found in KC patients can affect transcription, translation or have synergistic effect with other variants in causing the disease. It has been reported many times about synergistic effect of different mutations in mitochondria that can cause many severe diseases [38]. Nevertheless, it is impossible from the evolutionary point of view these sets of mutations to occur in the normal individual from different haplogroups. By keeping in mind about mutation rates in the coding region and its natural selection, we propose that these variants could theoretically influence the patient’s disease. Non-synonymous mutations and frame shift mutations adversely affect C1 synergetics resulting in increase ROS production and mitochondrial dysfunction. KC corneas are unable to process ROS due to depleted or low ATP levels and increased ROS production and thereby undergo oxidative damage. These corneas have increased levels of malondialdehyde (MDA), which can results in altered protein function leading to cascade of events, including apoptosis that can damage the corneal tissues.

Thus this pilot study highlights the role of sequence variation in mitochondrial complex I gene in keratoconus patients. Such cases with elevated free radicals levels and oxidative damage to cornea may benefit immensely by antioxidant therapy.

### Conclusions

Keratoconus corneas are known to suffer from oxidative damage. It is important to analyze the cause of raised ROS levels. Hence in this study we analyzed mitochondrial complex I genes, as sequence variations in this gene complex are associated with depleted ATP and increased ROS levels. We found a higher number of non-synonymous sequence variations in KC patients in comparison to controls.
